# Does the Hfq Protein Contribute to RNA Cargo Translocation into Bacterial Outer Membrane Vesicles?

**DOI:** 10.3390/pathogens14040399

**Published:** 2025-04-21

**Authors:** Marisela Velez, Véronique Arluison

**Affiliations:** 1 Instituto de Catálisis y Petroleoquímica (CSIC), c/Marie Curie 2, Cantoblanco, 28049 Madrid, Spain; 2 Laboratoire Léon Brillouin, UMR 12 CEA/CNRS, Site de Saclay, 91191 Gif-sur-Yvette, France; 3 Université Paris Cité, UFR SDV, 35 Rue Hélène Brion, 75013 Paris, France

**Keywords:** small noncoding regulatory RNA (sRNA), Hfq RNA chaperone, functional amyloid, lipid–protein interaction, cardiolipin, atomic force microscopy (AFM), periplasm, outer membrane vesicle (OMV)

## Abstract

Gram-negative bacteria release outer membrane vesicles (OMVs) that deliver various molecules, including virulence factors, to interact with their host. Recent studies have suggested that OMVs may also serve as carriers for RNAs, particularly small regulatory noncoding RNAs (sRNAs). For these RNAs to function effectively, they typically require a protein cofactor, Hfq, known as an RNA chaperone. In previous work, using molecular imaging, Circular Dichroism CD, and InfraRed FTIR spectroscopies, we demonstrated that Hfq interacts with the bacterial inner membrane and forms pores, suggesting a possible role in translocating RNA from the cytoplasm to periplasm and then to OMVs. In this study, we expand on our previous findings and provide evidence that RNA molecules bind to the *Escherichia coli* inner membrane in an Hfq-dependent manner. Moreover, we show that the lipid nature, in particular the presence of a cardiolipin-rich domain, is crucial for this interaction. These results reveal a new aspect of RNA translocation through the inner membrane, for further packaging in OMVs, and underscore the importance of Hfq in this mechanism.

## 1. Introduction

One important factor that enables bacteria to respond to external signals is the presence of small regulatory noncoding RNAs (sRNAs) [1,2]. Typically around 100 nucleotides long, these sRNAs regulate gene expression at the post-transcriptional level by binding imperfectly to their mRNA target(s) [3]. Nearly all prokaryotic organisms contain sRNAs [4], and extensive research on Gram-negative bacteria has revealed their crucial roles in various biological processes, such as virulence and nutrient acquisition [5,6,7]. For example, in the context of iron homeostasis, an sRNA called RyhB has been identified as a regulator of iron metabolism, helping bacteria to survive in iron-limited environments, for instance, within an eukaryotic host [8]. The action of sRNAs in general involves interactions with specific regions near the 5′ end of their target mRNAs, particularly within the translation initiation area (RBS, Ribosome-Binding Site, and AUG initiation codon). [2,3]. These interactions can either block or enhance the binding of the 30S ribosomal small subunit [3,9]. Because of their imperfect annealing to mRNA, sRNA action relies on additional protein factors, such as Hfq and ProQ, which act as chaperones, facilitating the sRNA-mRNA interaction [10]. Hfq, for instance, has multiple RNA-binding surfaces that allow it to bind both the sRNA and the mRNA simultaneously [11,12,13,14]. This increases the chance of base pairing and the kinetics of annealing. Hfq can also unwind or melt secondary structures in both sRNA and mRNA [15], exposing regions that are otherwise inaccessible for pairing. By holding the RNAs close and exposing their complementary regions, Hfq increases the kinetics of annealing—meaning the RNAs find and pair with each other more quickly [15]. The mechanisms of Hfq activities, however, are quite varied. Hfq does not only aid in sRNA:mRNA binding but also contributes to mRNA degradation [16,17]. Conversely, Hfq may also prevent sRNA degradation in some cases [18,19,20,21]. Moreover, Hfq shapes the bacterial chromosome [22,23] and influences various mechanisms related to DNA, such as replication, mutagenesis, or genetic recombination [24,25,26]. The effects of Hfq- on DNA-related processes may either be direct or indirect (i.e., using an sRNA-based regulation), as shown, for example, in its effect on the mutagenesis rate [26,27].

Structurally, Hfq is composed of two regions. The N-terminal region (NTR) of *Escherichia coli* Hfq forms a hexameric toroidal structure, referred to as an Sm-core [28,29], with six C-terminal regions (referred to as CTR in this manuscript), each composed of 38 amino acid residues, extending outward from the central core [11,30,31]. The hexameric structure and the RNA annealing function of Hfq mainly arise from the Sm-NTR [12,30,32]. Until recently, the role and structure of *E. coli* CTR were not well understood. However, recent studies have shown that the CTR adopts an amyloid-like structure [33,34]. The amyloid proteins are distinguished by their high β-sheet content, with a typical structure commonly referred to as the cross-β structure, and they typically exhibit a fibrillar morphology [35,36]. While amyloids are often linked to diseases such as Alzheimer’s in humans [37], they can also possess beneficial properties in some cases, and are thus referred to as functional amyloids [38,39]. This is particularly true for bacterial amyloids, which serve crucial and advantageous functions for the prokaryotic cell [40,41]. Functional bacterial amyloids, including Hfq-CTR, are usually characterized by a high content of alanine, asparagine, and threonine residues, and this is the case for Hfq-CTR (see Section 2.2). While the role of Hfq-CTR in RNA annealing remains somewhat uncertain and appears unnecessary for most sRNA-mediated regulations [32], its absence affects specific sRNA-based processes in vivo [42,43,44,45,46]. Furthermore, this domain has been shown to stabilize the subunit interface of the hexamer [31,43,47].

One crucial aspect of Hfq functions, precisely due to its amyloid CTR region, is its interaction with the bacterial membrane [48,49]. This interaction was first noted in vivo through imaging techniques [50,51], although the exact nature of the interaction remained unclear. It was estimated that about 50% of Hfq molecules are found in close proximity to the inner membrane [50]. Several hypotheses have been proposed regarding how Hfq interacts with the membrane, including binding to a membrane-bound nuclease or a post-translational modification involving an oxidized C:18 lipid [51,52,53]. However, using a synthetic peptide, it has been shown that the C-terminal region of Hfq can directly interact with the membrane without needing an intermediary protein or post-translational modification [48], utilizing the inherent ability of amyloids to bind to lipid bilayers [54,55]. Molecular microscopy studies have confirmed that Hfq’s interaction with the membrane results in membrane deformation and rupture [48]. Moreover, it has been shown that Hfq porates the inner membrane [49,56], and that discovery raised the possibility that Hfq might be exported in the periplasmic space. Transmission electron microscopy (TEM) studies have corroborated the proposal that Hfq is probably present in the periplasm [50], although the high resolution needed to definitively locate the protein between the inner and outer membranes of Gram-negative bacteria has prevented a clear conclusion (estimates of periplasm thickness vary from 10 to 50 nm [57]).

A significant element of bacterial communication involves outer membrane vesicles (OMVs) [58]. OMVs are tiny spherical structures (~100 nm) released by Gram-negative bacteria formed via the blebbing of the outer membrane [59,60]. They are essential for interkingdom cell-to-cell communication, in particular during the host infection. OMVs indeed mediate both bacterial virulence and immune modulation in a host [58,61,62,63,64]. Derived from the bacterial periplasm and outer membrane (OM), the composition of OMVs mirrors that of the periplasm and OM. OMVs contain a range of periplasmic and cytoplasmic components, collectively referred as cargo, including proteins, sugars (fragments of peptidoglycan), and toxins [60,65]. Beyond proteins, RNAs have also been identified within OMVs, gaining recognition as newly identified cargo [66,67]. These OMV-RNAs include small regulatory RNAs, tRNA, and mRNAs, although noncoding RNAs have been the more extensively studied [68,69]. The primary function of OMVs is to transport various biomolecules to host cells or other bacteria [62]. The cargo within OMVs can thus be internalized in the eukaryotic cell, transferring bacterial signaling cargos to alter the host cell’s behavior in ways that benefit bacterial proliferation. The presence of Hfq in the periplasm [50] raised the question of whether it might be encapsulated within OMVs. Western blot analysis has confirmed that Hfq is present in OMVs [70], though the exact location of the protein, in the membrane or within the lumen of OMVs, remains unclear.

In this work, we expanded on our earlier findings by showing that the *E. coli* Hfq-C-terminal region interacts with microdomains of the bacterial inner membrane, in agreement with our previous report [70]. Importantly, we demonstrate that inserted Hfq-CTR aids in the anchoring of RNA into these domains of the membrane. The RNA-IM interaction could thus be pivotal for RNA-driven regulatory processes involving OMVs [71], and our analysis may reveal mechanisms used by bacteria to export RNA in order to influence the genes expression profiles of their host [71].

## 2. Materials and Methods

### 2.1. Chemicals

All chemicals were purchased from Sigma-Aldrich (Saint Louis, MO, USA) or Thermofisher scientific (Waltham, MA, USA).

### 2.2. Preparation of Hfq-CTR

The sequence of the Hfq-CTR peptide corresponds to residues 64 to 102 in the full-length *E. coli* Hfq protein. The Hfq-CTR peptide was chemically synthetized (Proteogenix, Strasbourg, France).

Its sequence was: SRPVSHHSNAGGGTSSNYHHGSSAQNTSAQQDSEETE. The peptide was reconstituted in water at a concentration of 20 mg/mL (pH = 5.0). At this concentration, the peptide exhibits self-buffering properties and retains its activity [72].

### 2.3. DsrA sRNA Sequence and Preparation

For our analysis, we focused on a region of the DsrA small noncoding RNA (DsrA standing for “downstream of the replication initiator A” [73]). DsrA adopts a structure comprising three stem–loops (SL1, SL2, and SL3) with a lengthy linker region between SL1 and SL2 (Linker 1). SL3 functions as a Rho-independent transcription terminator. SL2 is involved in a dynamic conformational shift, enabling it to interact with various mRNAs [74]. In this paper, we focused our analysis on the SL1 and Linker 1 region that enhances the translation of *rpoS* mRNA, encoding the stress sigma factor σ^S^ [9,15]. The sequence of this region, referred to as DsrA_core_ in this manuscript, was AACACAUCAGAUUUCCUGGUGUAACGAAUUUUUUAAG. Before use, the DsrA_core_ oligonucleotide (Eurogentec, Seraing, Belgium) at 1mM (expressed in nucleotides) in water was melted at 80 °C for 1 min and then slowly cooled down at 20 °C to allow proper folding. This RNA was coupled to a biotin at the 5′ end.

### 2.4. Preparation of Small Unilamellar Vesicles (SUVs)

The protocol used for SUV preparation (2.4) and AFM imaging (2.5) was previously described in Turbant et al. [49]. Briefly, the *E. coli* Polar Extract (EPE) lipids from Avanti Polar Lipids (Alabaster, AL, USA) were prepared at a concentration of 10 mg/mL using a chilled chloroform:methanol 1:1 (*v/v*) solvent mixture. The EPE lipids formed a thin film by the gradual removal of solvent under a stream of N_2_(g), followed by an additional drying step for 30 min under nitrogen. The rehydration of the dried lipids was carried out in SUV buffer (10 mM Tris pH 7.5 containing 100 mM NaCl), after which vesicles were generated by gentle mixing at room temperature over 30 min. To obtain uniform SUVs, the lipid mixture was extruded approximately 30 times through a 0.2 µm polycarbonate filter using a Mini Extruder (Avanti Polar Lipids).

### 2.5. AFM Imaging in Solution

The suspension of SUVs was diluted to 0.4 g/L in SUV buffer supplemented with 2 mM CaCl_2_ and deposited onto freshly cleaved mica for an incubation at 37 °C for one hour. Afterward, the surface was rinsed thoroughly ten times with SUV buffer to remove non-adhered vesicles. The formation and integrity of the supported bilayer were verified by AFM imaging prior to the addition of the peptide. The Hfq-CTR peptide was applied to the bilayer at a concentration of 0.2 g/L and incubated at room temperature for 30 to 60 min inside a sealed chamber to prevent evaporation. The excess of peptides was removed by ten rinses with SUV buffer, and imaging continued in the same buffer. Imaging was conducted using rectangular silicon nitride cantilevers (PNP-DB from Nanoworld) on an Agilent Technologies 5500 AFM system (Santa Clara, CA, USA), operated in the tapping mode with a 15 kHz resonance frequency and a 0.48 N/m spring constant. Before initiating scans, the system was allowed to thermally equilibrate for approximately 30 min. Between five and ten locations were imaged at varying resolutions to ensure reproducibility. Images were analyzed using the WSxM 5.0 software (www.wsxmsolutions.com) and Gwyddion 2.62 free SPM analysis software (https://gwyddion.net/).

The RNA stock was diluted 3 times in water and incubated on top of the bilayer, previously incubated with Hfq-CTR for 40 min, after which the sample was extensively rinsed with SUV buffer before imaging.

The solution of colloidal particles modified with streptavidin (Alexa Fluor™ 488 Streptavidin, 5 nm colloidal gold conjugate, from Invitrogen/Thermofisher) was diluted 8 times in SUV buffer and incubated over the surface of the modified bilayer for 30 min. The sample was then again extensively rinsed with SUV buffer before imaging.

## 3. Results

### 3.1. The Amyloid CTR Region of Hfq Binds to Membrane Microdomains

**Figure 1:**
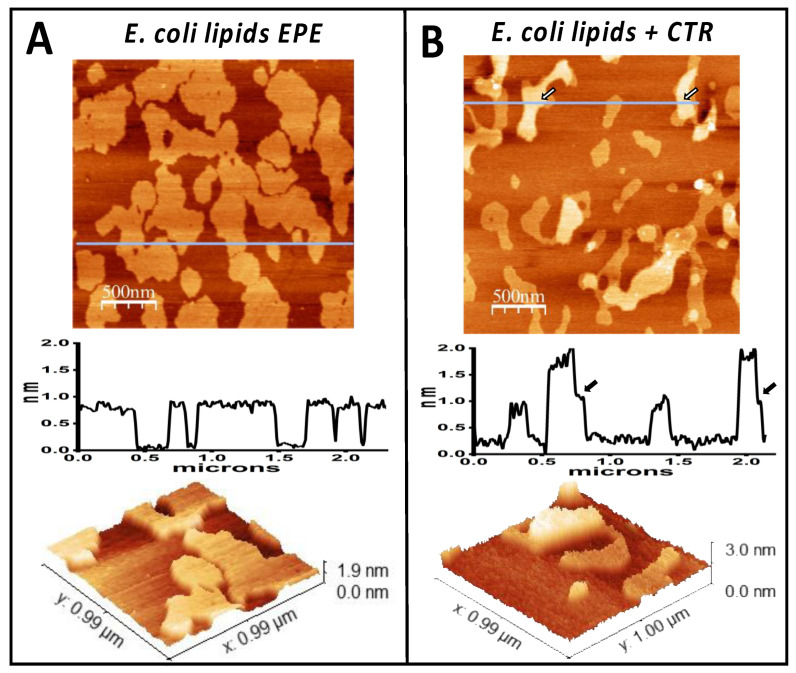
*E. coli* lipid bilayer incubated in the absence (**A**) or presence (**B**) of Hfq-CTR. Panel (**A**) shows the *E. coli* lipids bilayer. The height profile under the line shown on the upper image indicates that the domains are 0.8 nm higher than the rest of the membrane. The lower panel shows a three-dimensional representation of a small region. Panel (**B**) shows the *E. coli* lipid bilayer incubated in the presence of Hfq-CTR. The peptide accumulated on top of some of the domains, generating 1 nm high regions in some of them, as shown on the height profile. The arrows point the regions where the change in height occurs.

The *E. coli* inner membrane (IM) is mainly composed of four lipids: phosphatidylethanolamine (PE) (~75%), phosphatidylglycerol (PG) (~20%), cardiolipin (CL) (~5% during the exponential phase of growth, which can increase to 15–20% during the stationary phase [75,76]), and traces of phosphatidic acid (PA). To mimic the natural *E. coli* IM, we used EPE lipids extract containing 67% PE, 23% PG, and 10% CL. CL is a unique cone-shaped lipid and, due to its higher volume compared to the cylindrical shape of PG and PE and to its negative charge, it significantly influences membrane structure and dynamics [77]. While lipid rafts do not exist in bacteria, prokaryotes have equivalent membrane microdomains [78,79], and CL is the major component of these microdomains [80,81,82]. The conical shape of CL promotes its segregation, microdomain formation and facilitates negative membrane curvature [78]. Supported lipid bilayers of mixed composition including CL have been shown to present segregated domains that protrude around 1 nm from the rest. Figure 1A shows a supported lipid bilayer formed from the *E. coli* polar lipid extract. The domains observed are compatible with CL-rich microdomains [83]. Note that the lipids that interact the most with Hfq-CTR are phosphatidylglycerol (PG) and cardiolipin (CL—composed of two linked PG molecules [84]), whereas phosphatidylethanolamine (PE) and phosphatidic acid (PA) do not exhibit significant Hfq binding [56].

Figure 1B shows the membrane after exposure to Hfq-CTR for 45 min. A second layer of material was formed on top of some of the domains. These higher domains stabilize in about two hours into 1 nm high homogeneous and compact domains. Contrast in phase images obtained in tapping is associated with the mechanical properties of the sample. The different contrast observed between the higher and lower regions shown in Supplementary Figure S2 indicate that the presence of the protein could be rendering a certain stiffness to the bilayer. The fact that the phase images have a higher contrast in the higher regions indicates that these regions are mechanically more rigid than the bare lipids. Although this information is difficult to interpret at the molecular level, it does point to two possibilities: that the layer formed by the peptide is compact and, therefore, mechanically more rigid than the bilayer or that the peptide could be partially inserted into the membrane affecting its rigidity (as also described in [49]). It has been previously demonstrated that cardiolipin is the lipid that interacts most with Hfq-CTR [56], therefore reinforcing our hypothesis that the domains observed are enriched in cardiolipin. This hypothesis was strengthened by the preferred interaction of Hfq-CTR with these microdomains (Figure 1), as we previously demonstrated that cardiolipin is the lipid that interacts mostly with Hfq-CTR [56]. Note that a significant interaction between Hfq and PG also occurs, but in contrast to CL, PG is less prone to form membrane microdomains and is usually randomly distributed in the IM above 25 °C. We also observed that the domain size and shape may change over time after the interaction with Hfq-CTR, indicating that the lipid reordering might also by take place and that the composition of the domains might not be static.

### 3.2. The Amyloid CTR Region of Hfq Triggers RNA Insertion in the Memrbane

**Figure 2:**
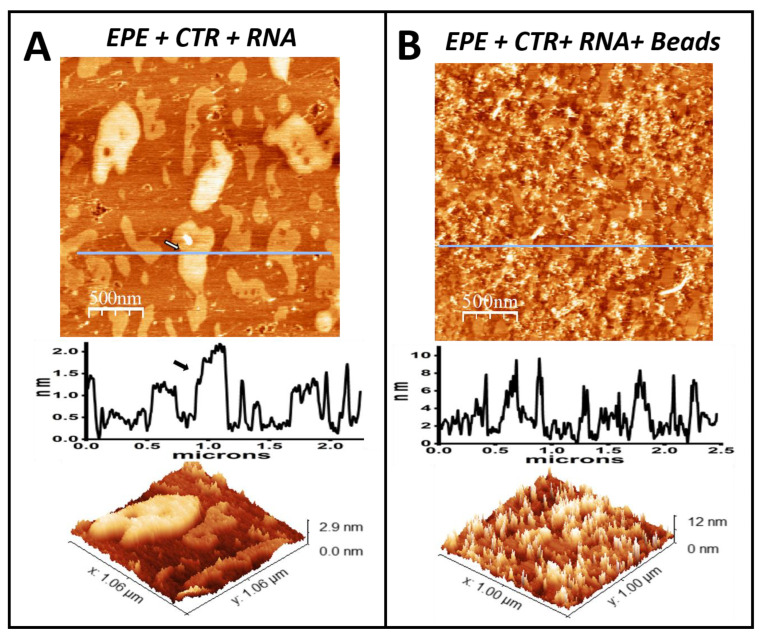
Effects of Hfq-CTR, RNA, and streptavidin gold beads on the *E. coli* lipid bilayer. (**A**) *E. coli* lipid bilayer (abbreviated as EPE) with HFq-CTR and RNA. The height profile under the line shown on the upper image indicates that some domain regions become higher, up to 1 nm above the domains, but no significant difference with respect to the bilayer prior to the incubation with RNA was detected. The arrows point the regions where the change in height occurs. The lower panel shows a three-dimensional representation of small region. (**B**) *E. coli* lipid bilayer incubated in the presence of Hfq-CTR, RNA, and streptavidin gold beads. The 5 nm diameter beads accumulate in some of the regions and the topographical profile detects reflects their presence.

The supported lipid membranes were incubated with RNA after their exposure to the Hfq-CTR peptide. Figure 2A shows that, as expected, the surface, although somewhat rougher, probably due to the presence of RNA, did not show any significant structural difference with respect to the one only containing the peptide. The higher regions lying on top of the lipid domains remained. When this modified surface was incubated for 30 min with 5 nm colloidal gold beads conjugated with streptavidin, the surface underwent significant remodeling. Figure 2B shows how the 5 nm beads accumulate preferentially in certain regions of the surface, which is perfectly compatible with their selective accumulation, on the peptide-enriched higher regions. The profile clearly indicated their presence on the surface. Figure S1 shows the two controls that indicate that both peptide and RNA are needed for the colloidal particles to attach to the lipids. When a membrane was exposed to Hfq-CTR and colloidal beads, as shown in Figure S1A, there was no attachment of colloidal particles. The same was observed when the membrane was only exposed to RNA before colloidal particles (Figure S1B). We therefore confirm that there is no non-specific binding to the membrane of the RNA nor the streptavidin-modified beads.

Indeed, the interaction between the *E. coli* membrane and RNA is not inherently favorable. Cationic lipids are not naturally present in this membrane and, in the absence of proteins, the *E. coli* inner membrane displays negative charges, which originate from the anionic lipids present in this membrane (PE, PG, and CL [85,86]). This likely explains why the presence of Hfq-CTR is necessary for RNA to bind to IM, as shown in this paper.

## 4. Discussion

**Figure 3:**
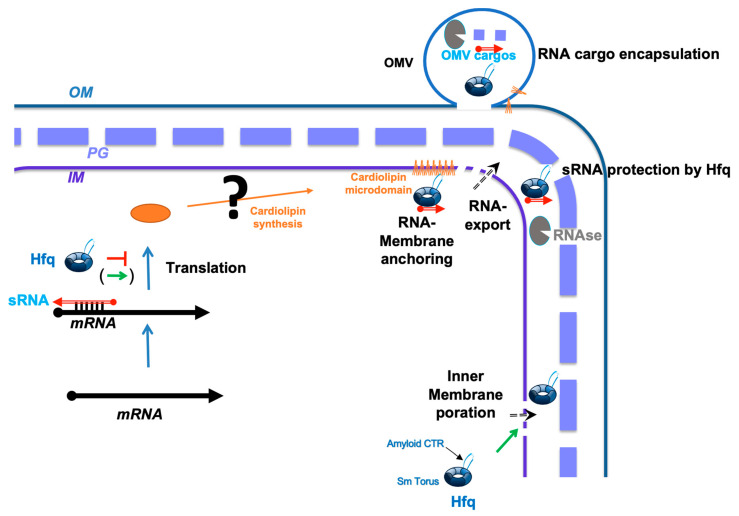
Model of Hfq-CTR interaction with *E. coli* membrane microdomain and possible consequence on OMV formation. Hfq interacts with CL-rich microdomains (in orange). As shown previously, this may promote hole formation and passive export. In this paper, we show that Hfq, and precisely its amyloid CTR, and sRNA bound to Hfq-CTR interact specifically with membrane microdomains. Hfq can protect sRNA from RNAse degradation in the periplasm and/or OMV lumen. sRNA regulators controlling mRNAs are depicted as red open arrows and mRNAs as thick black lines; 5′ and 3′ of RNAs are depicted by a “ball and arrow head”, respectively; Hfq-NTR is depicted as a blue toroidal hexamer (Sm torus); Hfq-CTR as a blue amyloid β-strand; CL synthases as orange ellipses and RNAses as grey ellipses; positive and negative regulations by Hfq are indicated by arrows and T-shaped lines, and colored in green and red, respectively; the dotted line symbolizes the peptidoglycans between the outer and inner membranes (OMs/IMs).

The presence of RNAs has been reported in OMVs from various Gram-negative bacteria [66,67,68,87]. Nevertheless, the mechanism by which these RNAs are packaged into OMVs remains unknown. Our previous studies suggested that these RNAs might be imported with the help of the Hfq chaperone, specifically using its CTR region, which physically interacts with the membrane and may allow the passive crossing of RNA through the IM to the periplasm [48,49]. In this paper, we show that Hfq-CTR is necessary for RNA to bind to the inner membrane, and this observation is compatible with RNA passing into the periplasm, where it can be encapsulated in OMVs (Figure 3). We have previously shown that Hfq is exported and present in the periplasm [50,70], and thus, believe that Hfq allows RNA periplasmic translocation for further export in OMVs. This mechanism indeed offers the advantage of protecting the RNAs from degradation both in the periplasm and within the OMV lumen, where RNases are abundant [19,88]. RNase I, for instance, mainly resides in the periplasm and could degrade unprotected periplasmic RNAs [89]. This association with Hfq could thus play an important role both in the translocation of the RNA through the inner membrane (using an unknown mechanisms), but also to prevent the premature degradation of the exported RNA immediately after its transit into the periplasm.

An important observation is the specific binding of Hfq or the Hfq:RNA complex to cardiolipin microdomains. CL is a unique, cone-shaped negative lipid, and its distinctive structure influences its propensity for phase transitions, with increasing membrane curvature, which affect vesicle budding [90,91,92]. This occurs mainly by CL sorting, which could be favored by Hfq binding. While cardiolipin is primarily found in the inner membrane, its presence in the *E. coli* outer membrane is also possible [93]. Thus, CL microdomains may attract Hfq and RNA to assist in their translocation within the periplasm, ultimately leading to embedding in OMVs (Figure 3). Another effect of CL, already reported for α-synuclein amyloid oligomers, is its ability to enhance pore formation in the inner mitochondrial membranes [94]. While in bacteria, CL has been proposed to prevent pore formation, this previous analysis did not use natural inner membranes and was conducted without a protein [95]. One can thus expect that, in addition to its role in OMV budding, CL attracts Hfq to promote the formation of pores in the inner membrane. Additionally, given Hfq’s role in RNA regulation, it is plausible that Hfq influences CL synthesis by modulating the expression or activity of enzymes involved in the CL biosynthetic pathway [96]. *E. coli* harbors three distinct enzymes that synthesize CL, ClsA, ClsB, and ClsC [97], and Hfq could influence their expression. Such a mechanism remains to be elucidated. Nevertheless, even if Hfq does not directly regulate cardiolipin synthases, it does regulate membrane stress responses and overall lipid homeostasis through the use of sRNAs. Hfq can thus potentially alter cardiolipin levels indirectly [96,98].

## 5. Conclusions

Although much is known about the passage of proteins across lipid bilayers, which often rely on precursors with a leader sequence for translocation through the membrane, data elucidating RNA translocation remain scarce. In this study, we present a model to better understand how Hfq might influence RNA translocation in the periplasm and its subsequent embedding into outer membrane vesicles. This work reveals potential mechanisms governing RNA transport and processing in bacterial systems, offering new insights into the interplay between RNA-binding proteins and membrane-associated pathways.

## Data Availability

The data that support the findings of this study are available upon request from the corresponding authors.

## Supplementary Materials

The following supporting information can be downloaded at: https://www.mdpi.com/article/10.3390/pathogens14040399/s1. Figure S1. (A) *E. coli* lipid bilayer incubated in the presence of CTR and streptavidin colloidal gold beads. The height profile corresponds to the line shown on the upper image. The lower panel shows a three-dimensional representation of a small region. When the membrane is incubated only with Hfq-CTR, colloidal beads do not accumulate on the surface and only the cardiolipin-enriched domains with the Hfq-CTR are observed; (B) *E. coli* lipid bilayer incubated in the presence of RNA and streptavidin colloidal gold beads. When the membrane is not incubated with Hfq-CTR prior to the addition of RNA, colloidal particles do not accumulate on the surface and only the cardiolipin-enriched domains are observed. Figure S2. Topographic and phase images of the lipid bilayer incubated with the Hfq-CTR peptide. The regions with peptide appear darker in the phase image, indicating that their mechanical properties are different. The peptide regions appear stiffer than the pure lipid regions.

## Abbreviations

The following abbreviations are used in this manuscript:

Outer membrane vesicle: OMV; IM/OM: Inner/Outer membrane; CL: Cardiolipin; AFM: Atomic force microscopy; Hfq-NTR/CTR: Hfq N/C-terminal region; EPE: *E. coli* polar extract; sRNA: Small noncoding RNA; PG: Phosphatidylglycerol; SUV: Small unilamellar vesicle.
