# Supplementary material for: Does the Hfq Protein Contribute to RNA Cargo Translocation into Bacterial Outer Membrane Vesicles?
Source: Pathogens. 2025 Apr 21;14(4):399. doi: 10.3390/pathogens14040399 (PMC12030562; doi:10.3390/pathogens14040399)
Supplement: Supplementary file 1 [file pathogens-14-00399-s001.zip › pathogens-3590656-supplementary.pdf]

Supplementary Material

Figure S1. (A) ***E. coli*** **lipid bilayer incubated in the presence of CTR and streptavidin colloidal gold beads.** The height profile corresponds to the line shown on the upper image. The lower panel shows a three dimensional representation of a small region. When the membrane is incubated only with Hfq-CTR, colloidal beads don´t accumulate on the surface and only the cardiolipin enriched domains with the Hfq-CTR are observed; **(B)** ***E. coli*** **lipid bilayer incubated in the presence of RNA and streptativin colloidal gold bead.** When the membrane is not incubated with Hfq-CTR prior to the addition of RNA, colloidal particles don´t accumulate on the surface and only the cardiolipin enriched domains are observed.

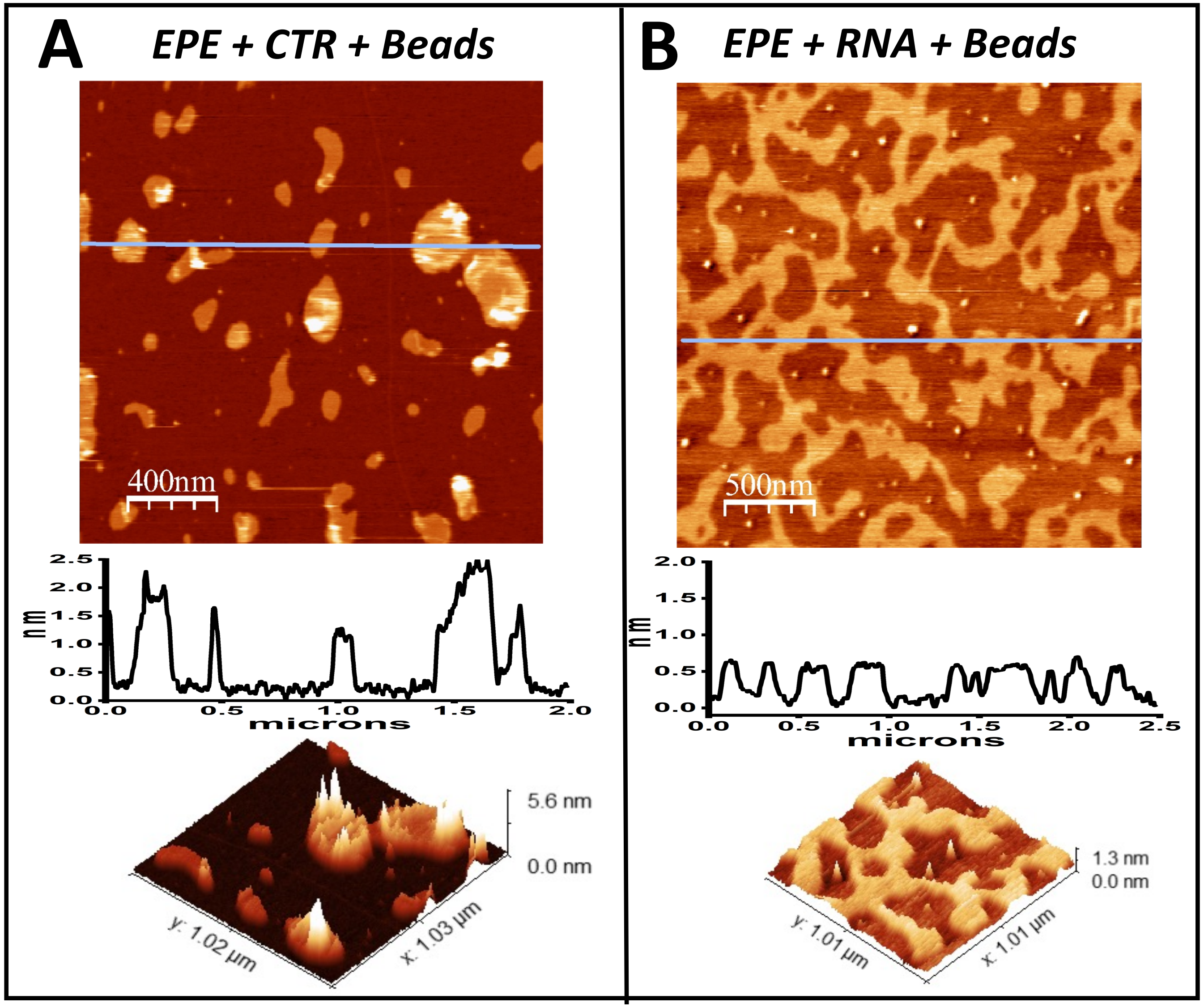

Supplementary Material

E. coli lipids EPE + CTR

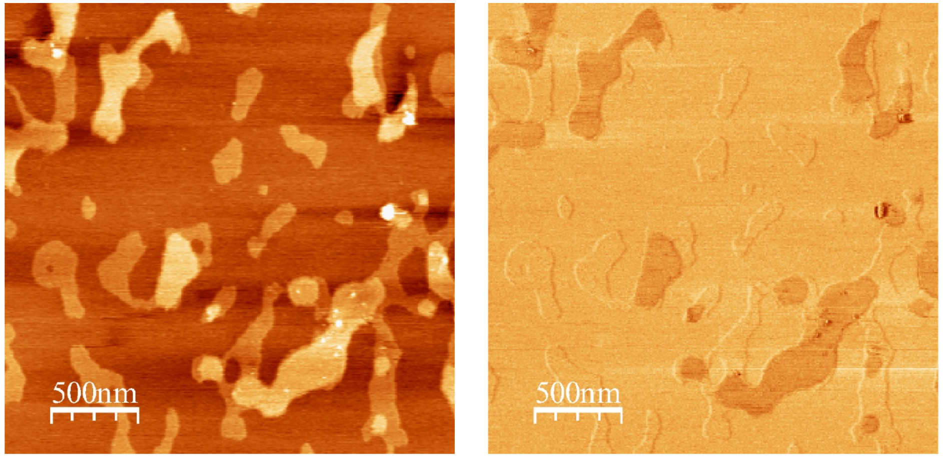

Topography **Phase**

Figure S2. Topographic and phase image of the lipid bilayer incubated with the Hfq-CTR peptide. The regions with peptide appear darker in the phase image, indicating that their mechanical properties are different. The peptide regions appear stiffer than the pure lipid regions.
